# Oocyte Maturation and Development

**DOI:** 10.12688/f1000research.7892.1

**Published:** 2016-03-09

**Authors:** Marie-Hélène Verlhac, Marie-Emilie Terret

**Affiliations:** 1 CIRB, Collège de France, Paris, France

**Keywords:** meiosis, gamete, zygotic development, oocyte, oocyte maturation

## Abstract

Sexual reproduction is essential for many organisms to propagate themselves. It requires the formation of haploid female and male gametes: oocytes and sperms. These specialized cells are generated through meiosis, a particular type of cell division that produces cells with recombined genomes that differ from their parental origin. In this review, we highlight the end process of female meiosis, the divisions per se, and how they can give rise to a functional female gamete preparing itself for the ensuing zygotic development. In particular, we discuss why such an essential process in the propagation of species is so poorly controlled, producing a strong percentage of abnormal female gametes in the end. Eventually, we examine aspects related to the lack of centrosomes in female oocytes, the asymmetry in size of the mammalian oocyte upon division, and in mammals the direct consequences of these long-lived cells in the ovary.

## Introduction: definitions

**Figure 1.**
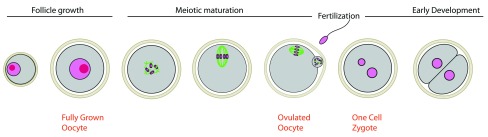
Meiotic maturation and first stages of embryo development in mammals. Meiotic maturation starts with nuclear envelope breakdown (NEBD) and is followed by the first meiotic division where bivalents are separated, the first polar body is extruded and then, in vertebrates, arrest in metaphase of the second meiotic division (MII stage) occurs. The oocyte is ovulated at the MII stage. Sister chromatids will be segregated after fertilization. Zygotic development follows fertilization. Oocytes (in gray) are surrounded by a protective glycoprotein layer, the zona pellucida (beige). DNA is in pink, microtubules in green.

All oocytes undergo induced arrest at the dictyate stage of prophase I during meiosis in the ovary. This arrest takes place after chromosome pairing and crossing-over formation between parental chromosomes. It can last months in mice and decades in humans. Upon hormonal surge, oocytes will exit the prophase I arrest and resume meiosis. All stages from meiosis resumption, starting with nuclear envelope breakdown (NEBD) until the next arrest where oocytes are fertilized, belong to the meiotic maturation process ( Figure 1). This process terminates meiosis, allowing the gamete to go through two successive rounds of extremely asymmetric divisions in size. Between these divisions, there is no intervening DNA replication, truly rendering the gamete haploid after, at, or before the second meiotic arrest at fertilization. Indeed, depending on the species, this second arrest will take place at different cell cycle stages: coincident with meiosis resumption at NEBD in nematodes, in metaphase I in *Drosophila*, in metaphase II for most vertebrates, or after the end of the second meiotic division in starfish oocytes.

There are three essential features that we would like to highlight in this review: (1) meiotic divisions take place in the absence of centrosomes in most animals; (2) these divisions are highly asymmetric in size to produce large oocytes; and (3) in mammals, oocytes display an extreme longevity in the ovary.

We would like to propose that these three features, among other things, predispose oocytes to errors in chromosome segregation. In mammals, this aspect has been particularly well studied, especially in human oocytes, which present a basal rate of errors close to 20% in women younger than 35 years of age and which can be as high as 60% in older women ^1– 3^. Indeed, it remains puzzling that given the extreme parental investment in juvenile care in many species, such little attention is being paid to oocyte ploidy, as if errors in chromosome segregation were part of a selection process for gamete fitness.

## Acentriolar divisions

**Figure 2.**
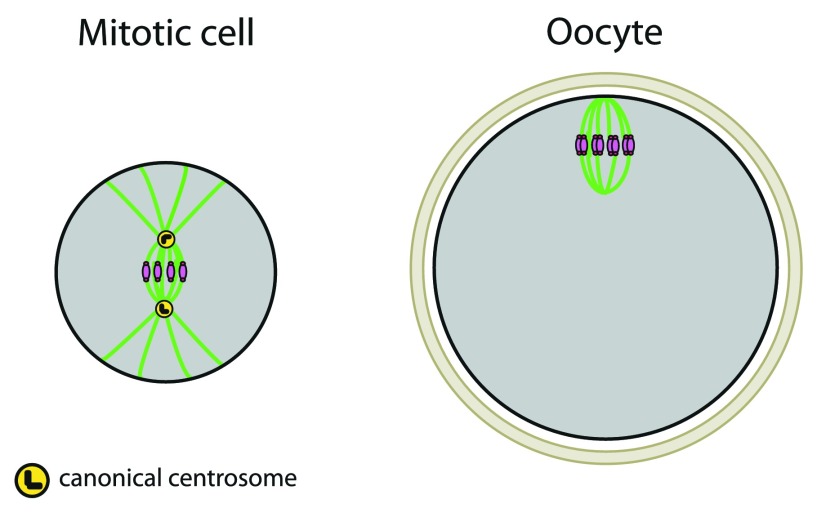
Oocytes assemble and position their spindle in the absence of centrosomes. Cells are in gray, and oocytes are surrounded by a protective glycoprotein layer, the zona pellucida (beige). DNA is in pink, microtubules in green, centrioles in black, and pericentriolar material (PCM) in yellow.

Centrosomes, consisting of a pair of centrioles surrounded by a cloud of pericentriolar material (PCM), are the major centers for microtubule assembly (microtubule-organizing center). Most oocytes lose their centrioles during oogenesis; if not, like in starfish, they are progressively eliminated and inactivated during meiotic divisions ^4– 8^. Even though centrosomes are not strictly required to segregate chromosomes (as shown in flies, planarians, or mice 9– 12), they contribute to the coordination of spindle assembly and increase its robustness. The immediate consequence of centrosome loss is that meiotic spindles are devoid of astral microtubules and so lack the main connector between the spindle poles and the cell cortex ( Figure 2). Hence, spindle positioning cannot rely on astral microtubules as it happens in most somatic cells ^13– 16^. Furthermore, centrosome-nucleated microtubules cannot capture chromosomes ^14^. In mitotic cells, duplicated centrosomes are positioned on opposite sides of the nucleus such that in prometaphase the spindle axis is already set ^17– 20^. On the contrary, meiotic spindle bipolarity is not predefined by the position of the two centrosomes. Instead, during meiosis I, spindle bipolarity is progressively established and this can take about 40 minutes in *Drosophila*, 3 hours in mouse, and up to 6.5 hours in human oocytes ^21– 25^. Not only is meiotic spindle bipolarity an extremely slow process in meiosis I but it appears that assembly of K fibers (microtubule bundles that connect the kinetochores of chromosomes) is quite long: 50 minutes in *Drosophila*, 6 to 7 hours in mice, and about 16 hours in humans ^26– 28^. The biological significance of such a progressive K fiber assembly during oocyte meiosis I remains unknown, but it is clear that in both *Drosophila* and mice precocious stabilization of K fibers is deleterious for bivalent alignment, orientation, and segregation ^28, 29^.

Oocytes use an inside/outside mode of spindle assembly, first promoting the assembly of microtubules around chromatin and then defining the spindle poles ^25, 30– 33^. As a result, meiotic spindle poles in oocytes appear less robust, not being anchored into unique and well-defined centrosomes. In some species, like *Drosophila*, nematodes, *Xenopus*, and even human oocytes, microtubule minus ends at spindle poles are not even connected or anchored to discrete PCM foci ^25, 34– 38^, unlike in rodents ^25, 34– 38^. The lack of anchoring raises issues not only on spindle pole organization and maintenance but also on the nature of the force integration that allows all chromosomes to end up midway in between both poles, on the metaphase plate. As a consequence of having poles formed by more than one entity, pole integrity can be compromised and splitting of poles may occur, as in cancer cells with extra-centrosomes presenting unbalanced poles composed of multiple centrosomes ^23, 31, 39^. It may not be so surprising that the rate of chromosome mis-segregation in oocytes is very high compared with most somatic cells in the presence of non-equilibrated spindle poles, which favor merotelic attachments not detected by the spindle assembly checkpoint (SAC) ^40^ as shown in cells with extra-centrosomes ^41, 42^.

In addition to lacking robust spindle poles, many oocytes use actin-based propulsion forces to position their chromosomes ^43– 50^. In starfish oocytes, an actin fishnet is transiently formed at meiosis resumption, prior to the microtubule capture, to maintain all chromosomes spatially confined, avoiding their dispersal in the huge volume of the nucleus, beyond the reach of microtubules ^51– 53^. In mitotic cells, even though astral microtubules dictate the orientation of the spindle apparatus, they are also connected to F-actin, which helps transmit forces exerted by the cell environing tissue ^54– 57^. In mitosis, microfilaments cooperate in spindle assembly: they help separate the two centrosomes in prometaphase, hence promoting spindle bipolarization ^58^, and they modulate spindle orientation and favor spindle assembly through mitotic cell rounding ^59^. However, in mammalian oocytes, where spindle bipolarization in meiosis I can take hours, it seems important that microfilaments do not interfere with spindle assembly and are thus nucleated around the microtubule spindle only once the latter is robust enough ^60^. It was very recently shown that the centrosome can also be a major filament-organizing center (it could be named an FTOC) ^61^. The centrosome thus acts as a coordinator of microtubule and microfilament networks inside the cytoplasm. In oocytes, this coordination is missing. Therefore, it is conceivable that other mechanisms have emerged to avoid premature interference between the two meshes and also to modulate their interaction.

## Extremely asymmetric divisions in size

**Figure 3.**
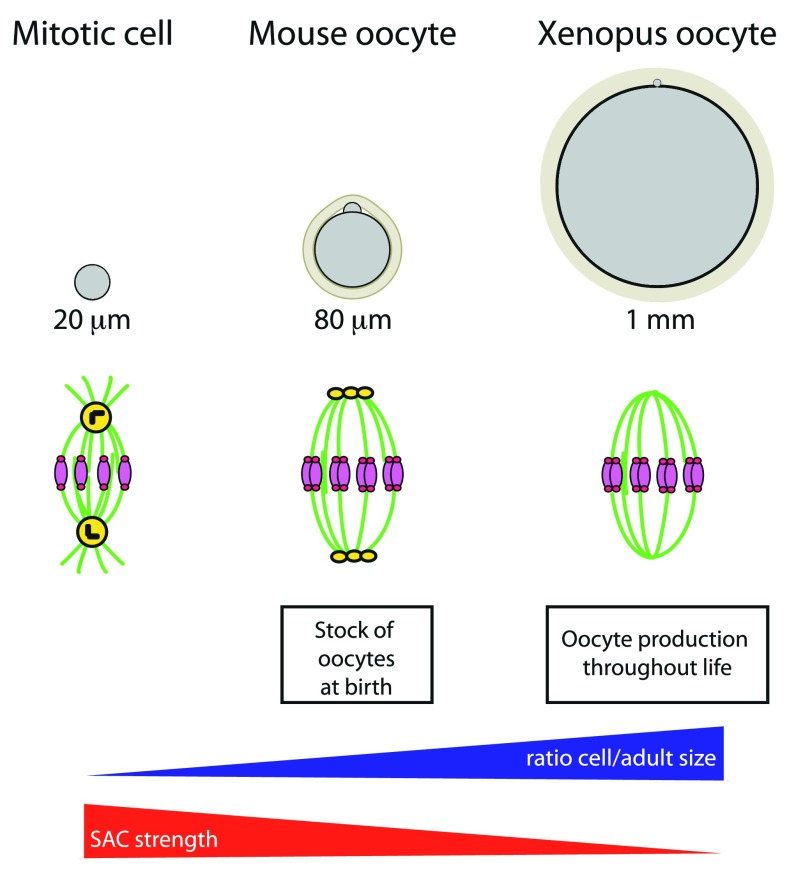
Spindle assembly checkpoint strength in different cells. Cells are in gray and oocytes are surrounded by a protective glycoprotein layer, the zona pellucida (beige). DNA is in pink, kinetochores in dark pink, microtubules in green, centrioles in black, and pericentriolar material (PCM) in yellow.

Oocytes undergo extremely asymmetric divisions, leading to the formation of a large cell, the oocyte, and two minuscule polar bodies. This size asymmetry is essential to maintain the maternal reserves accumulated during oogenesis in order to sustain embryo development. For this, they rely on very asymmetric spindle positioning. In the case of the *Xenopus* oocyte, 1 mm wide ( Figure 3), the asymmetry is clearly extreme where one spindle pole is anchored at the cortex while the other pole cannot reach the opposite cortex (the spindle being approximately 30 μm long). The asymmetric anchoring of the meiotic spindle to the cortex generates a strong imbalance of the forces experienced by each spindle pole, converted into asymmetric forces exerted on the chromosomes. How do oocytes achieve the equilibrium of tension on both sides of each bivalent (meiosis I) or univalent (meiosis II)? Moreover, when somatic cells enter mitosis, they round up and their cortical tension increases and this helps to equilibrate forces coming from each spindle poles to the chromosomes ^54, 62– 64^. Unexpectedly, mouse oocytes experience a drop in cortical tension during meiosis and this is absolutely necessary for spindle positioning as well as for the asymmetry of the division ^65– 67^. One can easily understand that a soft and deformable cortex favors the extrusion of polar bodies tailored to the chromatin mass better than a stiff cortex, as in mitosis. However, it is difficult to conceive how spindle microtubules can transmit and propagate the tension to chromosomes when their poles are not symmetrically anchored and when one pole is actually anchored on a soft material. One has to imagine that pushing or pulling forces might be transmitted more locally or maybe via yet-to-be-discovered structures/mechanisms inside the meiotic spindle. In worm oocytes, a solution has emerged with extensive meiotic spindle pole depolymerization at anaphase I and with most microtubule forces required to separate bivalent chromosomes coming from local microtubule assembly at the chiasmata, allowing the chromosomes to be pushed apart ^68^.

Another feature which characterizes oocytes is the poor sensitivity of the SAC to errors in chromosome alignment or to a global drop in tension exerted on bivalents ^69– 73^. In nematodes and *Xenopus* oocytes, there is no SAC response, and no cell cycle arrest is observed in *Caenorhabditis elegans* mutants with severe meiotic spindle defects or after complete microtubule depolymerization in the frog ^74– 77^. Similarly, mutations in multiple SAC genes do not affect cyclin B levels or chromosome segregation in *Drosophila* oocytes ^78^. In contrast, SAC-deficient *Drosophila* neuroblasts, genetically modified to lack centrosomes, present a higher incidence of chromosome segregation errors than acentrosomal neuroblasts with a functional SAC. This shows that, in *Drosophila* mitosis, a functional SAC is required, in the absence of centrosomes, contrary to what is observed in oocytes ^79^. Interestingly, all three of the above species assemble meiotic spindles without discrete PCM foci at their poles and this might contribute to the absence of a SAC response ( Figure 3).

As suggested by pioneering work from *Xenopus* early development, the origin of the poor SAC response in oocytes might come from their large size ( Figure 3) ^80^. The SAC signal, which inhibits the activation of the anaphase-promoting complex/cyclosome (APC/C) and thus the degradation of two key substrates, cyclin B and securin that trigger the metaphase-to-anaphase transition, is produced by unattached kinetochores and might be diluted in the large cytoplasmic volume. It will be very interesting to reduce oocyte size and see whether this restores a mitotic-like SAC response. Alternatively, it may not be strictly the oocyte size per se, but rather its size with respect to the dimensions of the adult female ( Figure 3). This ratio might relate better to the amount of energy invested by the species in its reproductive capacity. It could explain why, in nematodes and *Drosophila*, organisms a thousand times smaller than a mouse but producing eggs of comparable size as mammals, there is no SAC response during oocyte meiosis, as in *Xenopus* oocytes that lay eggs 12.5 times larger than mouse eggs for a comparable adult body size.

## Exacerbated longevity of mammalian oocytes

Thanks to a renewable population of germ cells that supports gametogenesis in their ovaries, nematodes, *Drosophila*, and amphibian females produce oocytes during their whole life. On the contrary, eutherian mammals possess a finite reserve of germ cells that are formed and stored during embryogenesis. The different reproductive strategies used by these model organisms might also explain the differences in SAC sensitivity ( Figure 3). Rapidly spawning a lot of eggs at the right season might have been selected to allow frog dissemination at the expense of gamete quality production. In contrast to species that lay eggs or embryos in the external milieu, in mammals, the longevity of oocytes from birth to ovulation can reach decades. This raises issues about chromatin architecture maintenance to sustain such a long-lived metabolism, in particular for the turnover of key elements involved in chromosome segregation. Meiotic spindle morphology is altered, with poor chromosome alignment and split poles in aging human oocytes obtained from normal naturally cycling women ^81^. Also, it has been clearly established that the amount of maternal mRNA encoding for genes involved in the SAC response, spindle integrity, and spindle positioning decreases in aged mouse oocytes ^82, 83^. More importantly, the number of proteins maintaining chromosome pairing reaches a critical low level in aged mouse and human oocytes, which impedes the integrity of chiasmata ^84– 86^. Furthermore, artificially abolishing one key meiotic cohesin, SMC1β, by gene targeting already has profound effects on the integrity of bivalents, and aged oocytes deficient for SMC1β display evidence of chiasma terminalization ^87^. The reduction in cohesin levels can be attributed to their lack of turnover. Indeed, genetic studies aimed at assessing the turnover of key cohesin subunits, such as the meiotic cohesin Rec8 or SMC1β, have demonstrated that these cohesins are not replenished after birth in the growing follicles of the mouse ^88, 89^. The progressive deterioration of cohesion could potentially be a leading cause for the increase in errors in chromosome segregation (in particular, errors in meiosis I) observed with age ^1, 3, 90^. Nonetheless, in *Drosophila* oocytes, evidence for cohesin rejuvenation during oogenesis has been shown ^91^. Whether this aspect is specific to *Drosophila* or can be extended to mammals is currently unknown.

The effect of cohesin deterioration can be further amplified by the poor sensitivity of the SAC, at least in mice, to a reduction in tension on bivalents ^71^. Recently, direct observations made on human oocytes have shown that sister kinetochores tend to split prematurely during meiosis I and that this effect increases with age ^92, 93^. Premature splitting potentially favors precocious bivalent dissociation into univalent, contributing to aneuploidy.

## Conclusions

Amazing progress has been made since the pioneering review that put into the limelight the fact that human oocytes are error prone and that the rate of errors increases with the age of the mother ^1^. This review challenged scientists in the field to try to understand a major societal issue of our modern societies where women postpone childbearing and to which female scientists were maybe already particularly exposed. Advance has come from studies on diverse model systems presenting both similarities and differences with human oocyte meiosis. Importantly, observations made in human oocytes have been challenged by hypotheses tested in model systems where genetics or biochemistry can be performed. Obviously, it is a combination of factors that seem to predispose oocytes to aneuploidy: their confounding fragile mode of spindle assembly and positioning, coupled to the pressure to preserve maternal stores in a gigantic cell via extreme asymmetry in size of their divisions as well as their longevity. Of course, many other factors not highlighted here, such as the distribution as well as the rate of recombination between homologues, which have a direct influence on chromosome segregation, can also predispose for aneuploidy in oocytes ^3^. The major discovery of PRDM9, a key factor controlling the distribution of hot spots for recombination in some species but not others, will certainly help us to understand the impact of recombination on aneuploidy in oocytes ^94– 96^. Indeed, meiotic maturation is embedded in a continuous process that started in the embryo and that resumes later in the life of an animal. Meiotic maturation also prepares the gamete for fertilization and early development; hence, gamete quality does greatly influence the chances of a successful pregnancy.
